# Survivors of acute kidney injury requiring renal replacement therapy rarely receive follow-up: identification of an unmet need

**DOI:** 10.1186/cc12365

**Published:** 2013-03-19

**Authors:** CJ Kirwan, R Taylor, JR Prowle

**Affiliations:** 1 The Royal London Hospital, Barts Health NHS Trust, London, UK

## Introduction

Acute kidney injury (AKI) occurs in more than 50% of ICU admissions, requiring renal replacement therapy (RRT) in around 10% of cases. There is now increasing evidence that AKI is a risk factor for the development and progression of chronic kidney disease (CKD); however, when AKI occurs as a complication of critical illness appropriate follow-up may be neglected. Accordingly, we reviewed the follow-up of renal function in all patients who received RRT on our ICU and survived to hospital discharge.

## Methods

A retrospective audit of patients who received RRT in a central London adult critical care unit during 2011.

## Results

Of 921 patients admitted, 203 received RRT with 109 surviving to hospital discharge. We excluded 52 patients who had end-stage renal disease, renal transplant or known glomerular disease. Of the remaining 57 AKI patients, median age was 60 (range: 18 to 77) and 37 (65%) were male. Median discharge creatinine was 74.5 μmol/l (27 to 662). Forty-two (74%) were offered follow-up, but in only six cases (11%) was this to nephrology services. Twenty-eight attended follow-up (five to nephrology) at a median time of 6 weeks; however, creatinine was measured at in only 14 and in six of these it had risen (by median 16.5 μmol/l). In addition, 14 patients had creatinine measured 3 to 6 months post discharge and in eight it had risen (by median 31.5 μmol/l).

## Conclusion

**Figure 1:**
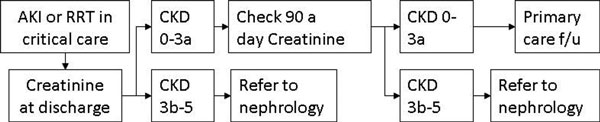
**A suggested follow-up pathway for AKI survivors**.

Follow-up of patients who received RRT for AKI in the ICU was poor and they were rarely referred to nephrologists. Where renal function was measured after discharge, there was evidence of progressive renal dysfunction; however, renal function was often not assessed. We propose an algorithm for clinicians to guide follow-up. See Figure 1.

